# Perceived Exertion in Physical Activity Measurement Across the Lifecourse: Results from SWAN

**DOI:** 10.1093/geroni/igab046.3254

**Published:** 2021-12-17

**Authors:** Kelly Ylitalo, Carrie Karvonen-Gutierrez, Barbara Sternfeld, Kelley Pettee Gabriel

**Affiliations:** 1 Baylor University, Waco, Texas, United States; 2 University of Michigan, School of Public Health, Ann Arbor, Michigan, United States; 3 Kaiser Permanente, Oakland, California, United States; 4 The University of Alabama at Birmingham, Birmingham, Alabama, United States

## Abstract

Physical activity (PA) guidelines recommend 150 minutes of moderate-intensity or 75 minutes of vigorous-intensity PA, in addition to muscle strengthening activities, each week. Many questionnaires ascertain PA frequency, duration, and intensity to benchmark achievement of PA recommendations. However, most scoring algorithms utilize absolute intensity estimates when exertion may be influenced by age or other sociodemographic or health characteristics. This study compared PA estimates with and without adjustments for perceived exertion and determined if that difference was associated with individual characteristics. Women (n=2,711) from the longitudinal Study of Women’s Health Across the Nation who completed ≥3 Kaiser Physical Activity Surveys (KPAS) across 8 biennial visits were included (baseline age: 46.4±2.7 years). KPAS responses were converted to metabolic equivalent of a task (METs) using the Compendium of Physical Activities to estimate absolute and perceived intensity-adjusted MET values. Latent class growth modeling identified subgroups of participants following similar patterns of change in the difference between absolute intensity-based and perceived intensity-adjusted estimates across time. Four major trajectory classes emerged with patterns reflecting: (1) lessening high-intensity exercise (2.2%); (2) increasingly high-intensity exercise (3.1%); (3) consistently moderate-intensity exercise (92.0%); and (4) consistently low-intensity exercise (2.8%). Consistently low-intensity exercisers, for whom absolute intensity-based estimates exceeded perceived intensity-adjusted estimates, were more likely to be Japanese or Chinese (p<0.001) and have lower BMI (p=0.05). However, for most participants, absolute intensity-based estimates approximated perceived intensity-adjusted estimates over time, suggesting that traditional PA scoring techniques may provide sufficient estimates for PA in longitudinal cohort studies of mid-life and older adult women.

